# Detection of *Leishmania* RNA Virus 1 in *Leishmania (Viannia) panamensis* Isolates, Panama

**DOI:** 10.3201/eid2906.220012

**Published:** 2023-06

**Authors:** Kadir Gonzalez, Santiago S. De León, Vanessa Pineda, Franklyn Samudio, Zeuz Capitan-Barrios, José Antonio Suarez, Adriana Weeden, Betsi Ortiz, Margarita Rios, Brechla Moreno, Nathan D. Gundacker, Juan M. Pascale, Sandra López-Vergès, Néstor Sosa, Azael Saldaña, Leyda E. Ábrego

**Affiliations:** Gorgas Memorial Institute for Health Studies, Panama City, Panama (K. González, S.S. De León, V. Pineda, F. Samudio, Z. Capitan-Barrios, J.A. Suárez, A. Weeden, B. Ortíz, M. Ríos, B. Moreno, J.M. Pascale, S. López-Vergès, N. Sosa, A. Saldaña, L.E. Ábrego);; University of Panama, Panama City (K. Gonzalez, S.S. De León, Z. Capitan-Barrios, A. Saldaña, L.E. Ábrego);; Medical College of Wisconsin—Zablocki VA Medical Center, Milwaukee, Wisconsin, USA (N.D. Gundacker);; University of New Mexico, Albuquerque, New Mexico, USA (N. Sosa)

**Keywords:** Leishmania, Leishmania RNA virus 1, Leishmaniavirus, LRV1, Leishmania (Viannia) panamensis, viruses, parasites, cutaneous leishmaniasis, mucocutaneous leishmaniasis, Panama

## Abstract

We detected *Leishmania* RNA virus 1 (LRV1) in 11 isolates of *Leishmania (Viannia) panamensis* collected during 2014–2019 from patients from different geographic areas in Panama. The distribution suggested a spread of LRV1 in *L. (V.) panamensis* parasites. We found no association between LRV1 and an increase in clinical pathology.

Leishmania RNA virus 1 (LRV1) belongs to the *Totiviridae* family, *Leishmaniavirus* genus, and infects different *Leishmania* lineages. This virus is not enveloped and is composed of a viral capsid ≈40 nm in diameter and a double-stranded RNA (dsRNA) of 5,280 nt (*1*,*2*). The genome has 3 open reading frames (ORF), 2 of which are coding. The *orf2* codes for the capsid protein and the *orf3* codes for an RNA-dependent RNA polymerase (RdRp). *orf1* has been described in other members of the family, but its function is unknown (*1*,*3*). This virus has been categorized in LRV1 and LRV2, according to the subgenuses of *Leishmania* in which they have been identified (*4*,*5*). The presence of LRV1 has been reported more frequently in specific regions of South America associated with cases of cutaneous leishmaniasis (CL) and mucocutaneous leishmaniasis (MCL) (*6*,*7*). *L. (Viannia) panamensis* is the predominant species and is responsible for most cases of CL in Panama (*8*,*9*) and the presence of LRV1 has been reported in 2 isolates of *L. (V.) panamensis* from Ecuador and Costa Rica (*7*,*10*). 

## The Study

We analyzed *Leishmania* spp. parasite isolates from clinical samples from 2014–2018 that were cryopreserved at Gorgas Memorial Institute’s parasitology research department (Panama City, Panama). The Bioethics Committee of the Gorgas Memorial Institute for Health Studies approved this study (protocol no. 056/CBI/ICGES/19). We extracted clinical and epidemiologic data such as sex, age, clinical classification (location, severity, and number of lesions), and province of origin from the database. The disease was classified as nonsevere or severe according to Infectious Disease Society of America guidelines (*11*). We activated the isolates at 26°C by using Schneider’s medium enriched with 25% fetal bovine serum until reaching exponential growth (2–3 ×10^7^ parasites/mL) (*9*). We centrifuged this concentration of parasites for 10 minutes at 3,500 rpm and divided it into 2 pellets; we used 1 pellet to extract DNA from *Leishmania* spp. for characterization and confirmation and the other to extract RNA and detect LRV1. We characterized the isolates as *L. (V.) panamensis* by the RFLP/PCR-Hsp70 methodology (*12*). For the detection of LRV1, we amplified 245 nucleotides corresponding to the *orf1* gene region using the primers described by Ito et al. (*6*,*13*) and sequenced the product by the Sanger method.

**Figure 1:**
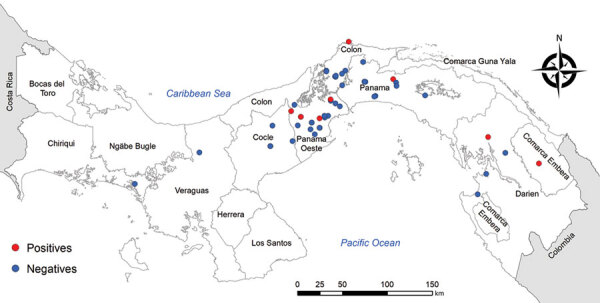
Distribution map of Leishmania RNA virus 1 positive and negative  isolates analyzed in Panama, 2014–2018.

We recovered parasite isolates from 56 patients. Of those isolates, 11 (20%) were positive for LRV1, 63.3% from female patients and 36.4% from male patients. Patient age range was 8–59 years; mean (+SD) age was 34 (+5.4) years (Appendix Table 1). All the patients came from leishmaniasis-endemic areas in Panama: 36.4% from Panama Oeste, 18.2% from Panama, 18.2% from Colón, 18.2% from Darién, and 9.0% from Coclé (Figure 1). Most of the patients had single lesions (7/11 [63.6%]); mean (+SD) was 1 (+0.2) and range 1–3 lesions per patient. Mean (+SD) time of evolution of the lesion was 50 (+9.6) days and range was 21–120 days. Most (6/11 [54.5%]) patients had an evolution time of 30 days. All the lesions were CL and were classified as nonsevere; lesions consisted of a crusty, moist ulcer with raised margins and a clean base (Table) (*11*). The lesions were distributed mainly on the arms (9/11: 81.8%); only 2 were visible elsewhere, on the leg (1/11: 9.1%) and face (1/11: 9.1%).

**Table:** Epidemiologic description of *Leishmania (Viannia) panamensis* isolates analyzed for LRV1, Panama, 2014–2018*

LRV1 status	No. isolates, N = 56	Mean age, y (SD)	Age range, y	Sex, no.	Duration of disease, d			No. lesions	
					Mean (SD)	Range		Mean (SD)	Range
Positive	11	34 (5.4)	8–59	4 M, 7 F	50 (9.6)	21–120		1 (0.2)	1–3
Negative	45	30 (3.1)	3–72	26 M, 19 F	67 (11)	15–365		1 (0.2)	1–6

*LRV1,* Leishmania *RNA virus 1.

**Figure 2:**
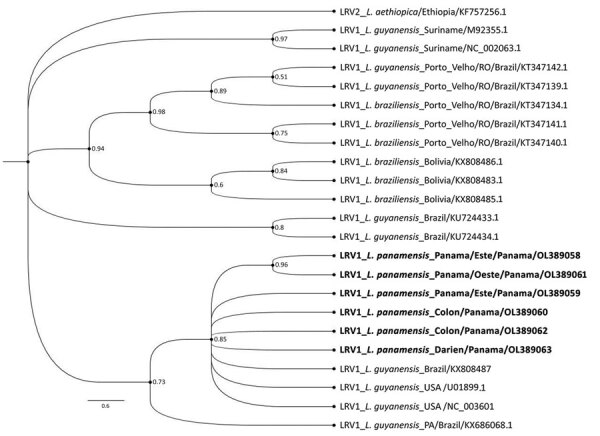
Phylogenetic analysis of *Leishmania* RNA virus 1 isolates analyzed in Panama, 2014–2018, and reference isolates. A phylogenetic tree reconstruction was implemented, applying Bayesian inference with the general time reversible plus gamma 4 plus invariate sites model using MrBayes version 3.2.6 phylogenetic software (https://nbisweden.github.io/MrBayes). Boldface indicates sequences obtained in this study, which are in the same clade with reference sequences from *Leishmania (Viannia) panamensis* isolates, mostly from Brazil. Numbers at each node represent clade credibility values. GenBank accession numbers are provided. Scale bar indicates substitutions per site.

We performed data analysis using GraphPad Prism 5.0 software (GraphPad, https://www.graphpad.com). We performed the Kolmogorov-Smirnov test to assess the normality of the samples. To analyze the differences between groups, we performed a *t* test for Gaussian distribution data. We considered differences statistically significant when p was <0.05. We found no significant difference to suggest that those with LRV1-positive parasites developed more severe diseases (data not shown). From 10 sequences obtained in the study (GenBank accession nos. OL389058–67), we selected 6 sequences based on phylogenetic analysis quality (Appendix Table 2); those sequences clustered within the phylogenetic group of LRV1 sequences detected in the species of the subgenus *Viannia*, close to those found in isolates of *L. (V.) guyanensis* (Figure 2).

## Conclusions

We detected LRV1 in 11/56 (20%) of *L. (V.) panamensis*–evaluated isolates, all of them in patients with CL, consistent with the preliminary description of the presence of LRV1 in 2 isolates of *L. (V.) panamensis* from clinical samples from Ecuador and Costa Rica, countries geographically close to Panama (*7*). The prevalence of LRV1 has been reported as higher in *Leishmania* spp. isolates from the New World (39.1%) than in those from the Old World (8.4%); prevalence also is higher in isolates from patients with severe skin forms of leishmaniasis, such as disseminated leishmaniasis and MCL, than from patients with CL (*14*). 

The use of *Leishmania* spp. isolates could be a limitation for the analysis because we were able to analyze only the parasites that grew in medium. To avoid this bias, future studies analyzing the presence of the virus directly from clinical samples are needed. In South American countries, prevalence of ≈25% of LRV1 has been described in isolates of *L. (V.) braziliensis* and *L. (V.) guyanensis* from Peru (*7*), Bolivia (*14*), and Brazil (*15*). The presence of LRV1 in *L. (V.) panamensis* in this study (20%) indicates circulation of this virus in Panama, suggesting LRV1 is likely widespread across the Americas and in different *Leishmania (V.)* species. Future analysis using a higher number of samples is necessary to estimate LRV1 prevalence in Panama.

In this study, we found no evidence that correlates the presence of LRV1 with severe clinical forms of leishmaniasis caused by *L. (V.) panamensis*, which was consistent with previous findings of no predisposition of the Th2 response induced by LRV1 for the favorable survival of the parasite for *L. (V.) panamensis* (*7*). In addition, previous studies described a general decrease in the expression of virulence factor transcription in *L. (V.) panamensis* (*7*) compared with an earlier study of *L. (V.) braziliensis* (*10*). It is possible that *L. (V.) panamensis* strains infected with LRV1 have low expression of virulence factor, which would be reflected in the presence of uncomplicated symptoms of CL cases in the analyzed samples.

The role of LRV1 and its subtypes modulating the immune response in infection caused by *L. (V.) panamensis* is unclear. It is important to carry out studies of the virus subtypes that are circulating in the country and analyze whether the differences in the modulation of the immune response reflected in the clinical manifestations are because of intrinsic factors of the virus, the *Leishmania* species that it infects, or both.

In conclusion, the data we obtained show the presence of LRV1 in isolates of *L. (V.) panamensis* from Panama from different years and locations, suggesting wide spread of the virus in this species. In addition, the recent documented circulation of *L. (V.) guyanensis* and *L. (V.) braziliensis* in Panama (*9*) and the proposed association of LRV1 presence in these species with severity of disease highlight the necessity of future studies on the presence of LRV1 in non–*L. (V.) panamensis* species in Panama. The role of *Leishmania* in disease severity may depend on the species infected and the role of viral, parasite, and human host factors in pathogenesis.

Appendix Additional information about *Leishmania* RNA virus 1, Panama.
