# Supplementary material for: Detection of Leishmania RNA Virus 1 in Leishmania (Viannia) panamensis Isolates, Panama
Source: Emerg Infect Dis. 2023 Jun;29(6):1250–3. doi: 10.3201/eid2906.220012 (PMC10202884; doi:10.3201/eid2906.220012)
Supplement: Appendix — Additional information about Leishmania RNA virus 1, Panama. [file 22-0012-Techapp-s1.pdf]

Article DOI: https://doi.org/10.3201/eid2906.220012

EID cannot ensure accessibility for supplementary materials supplied by authors. Readers who have difficulty accessing supplementary content should contact the authors for assistance.

Detection of *Leishmania* RNA Virus 1 in *Leishmania (Viannia) panamensis* Isolates, Panama

Appendix

Appendix Table 1. Clinical and epidemiologic description of the 56 *Leishmania (V.) panamensis* isolates analyzed in this study

ID	Province of origin	Age, y	Sex	Duration of disease, d	No. lesions	LRV1 status	GenBank accession no.
1	Panama	8	F	60	1	Positive	OL389058
2	Panama	46	F	21	1	Positive	OL389059
3	Colon	41	M	90	1	Positive	OL389060
4	Panama Oeste	17	M	60	1	Positive	ND
5	Panama Oeste	59	F	21	3	Positive	OL389064
6	Darien	57	F	30	2	Positive	OL389065
7	Panama Oeste	20	F	60	1	Positive	OL389066
8	Panama Oeste	51	F	30	1	Positive	OL389061
9	Cocle	14	F	120	2	Positive	OL389067
10	Colon	39	M	42	1	Positive	OL389062
11	Darien	31	M	21	2	Positive	OL389063
12	Panama Oeste	44	M	30	1	Negative	ND
13	Chiriqui	9	M	90	1	Negative	ND
14	Panama Oeste	25	M	30	4	Negative	ND
15	Veraguas	17	F	60	2	Negative	ND
16	Colon	18	M	90	1	Negative	ND
17	Panama	30	M	120	1	Negative	ND
18	Panama	23	F	90	1	Negative	ND
19	Darien	40	F	30	1	Negative	ND
20	Cocle	12	M	21	1	Negative	ND
21	Colon	8	M	20	1	Negative	ND
22	Darien	17	F	120	2	Negative	ND
23	Panama	48	M	42	5	Negative	ND
24	Panama	35	F	ND	4	Negative	ND
25	Darien	38	M	360	1	Negative	ND
26	Panama	9	F	30	1	Negative	ND
27	Panama	ND	F	ND	1	Negative	ND
28	Panama Oeste	52	F	30	1	Negative	ND
29	Darien	64	M	15	6	Negative	ND
30	Panama	63	M	365	1	Negative	ND
31	Panama	65	M	21	1	Negative	ND
32	Colon	54	F	30	2	Negative	ND
33	Panama	4	M	30	1	Negative	ND
34	Panama Oeste	20	F	42	1	Negative	ND
35	Cocle	30	M	60	2	Negative	ND
36	Colon	18	M	30	1	Negative	ND
37	Panama Oeste	15	F	60	2	Negative	ND
38	Panama Oeste	14	M	30	2	Negative	ND
39	Cocle	7	M	30	1	Negative	ND
40	Panama Oeste	20	M	21	1	Negative	ND
41	Colon	3	F	60	1	Negative	ND
42	Panama Oeste	28	F	60	1	Negative	ND
43	Panama Oeste	19	M	30	1	Negative	ND
44	Panama Oeste	15	M	60	1	Negative	ND
45	Panama Oeste	9	F	45	2	Negative	ND
46	Panama Oeste	69	F	60	2	Negative	ND

ID	Province of origin	Age, y	Sex	Duration of disease, d	No. lesions	LRV1 status	GenBank accession no.
47	Panama Oeste	14	M	90	1	Negative	ND
48	Panama Oeste	41	M	23	3	Negative	ND
49	Panama Oeste	36	F	60	2	Negative	ND
50	Cocle	48	F	90	2	Negative	ND
51	Colon	20	F	60	1	Negative	ND
52	Panama Oeste	16	M	60	3	Negative	ND
53	Cocle	54	F	90	2	Negative	ND
54	Colon	53	M	45	1	Negative	ND
55	Panama	72	M	60	1	Negative	ND
56	Colon	12	M	90	2	Negative	ND

*LRV1, Leishmania RNA virus 1; ND: data not available

Appendix Table 2. Sequence quality and access codes for the GenBank of the *Leishmania. (Viannia) panamensis* isolates positive to LRV1

Strain ID	Parasite	Leishmania International code	Sequence length, bp	GenBank accession no.
C-16-34	*L. (V.) panamensis*	MHOM/PA/2016/C1634	214	OL389058*
C-16-72	*L. (V.) panamensis*	MHOM/PA/2016/C1672	211	OL389059*
C-16-78	*L. (V.) panamensis*	MHOM/PA/2016/C1678	212	OL389060*
C-18-151	*L. (V.) panamensis*	MHOM/PA/2018/C18151	158 (Forward)	OL389064
C-18-163	*L. (V.) panamensis*	MHOM/PA/2018/C18163	143 (Forward)	OL389065
C-18-167	*L. (V.) panamensis*	MHOM/PA/2018/C18167	147 (Reverse)	OL389066
C-18-176	*L. (V.) panamensis*	MHOM/PA/2018/C18176	214	OL389061*
C-18-185	*L. (V.) panamensis*	MHOM/PA/2018/C18185	146 (Forward)	OL389067
C-18-186	*L. (V.) panamensis*	MHOM/PA/2018/C18186	215	OL389062*
C-18-196	*L. (V.) panamensis*	MHOM/PA/2018/C18196	212	OL389063*

*Sequences used in the phylogenetic analysis
